# Brief Report: HIV Incidence Among Older Adults in a Rural South African Setting: 2010–2015

**DOI:** 10.1097/QAI.0000000000002404

**Published:** 2020-06-02

**Authors:** F. Xavier Gómez-Olivé, Brian Houle, Molly Rosenberg, Chodziwadziwa Kabudula, Sanyu Mojola, Julia K. Rohr, Samuel Clark, Nicole Angotti, Enid Schatz, Kathleen Kahn, Till Bärnighausen, Jane Menken

**Affiliations:** a MRC/Wits Rural Public Health and Health Transitions Research Unit (Agincourt), School of Public Health, Faculty of Health Sciences, University of the Witwatersrand, Johannesburg, South Africa;; b School of Demography, the Australian National University, Canberra, Australia;; c CU Population Center, Institute of Behavioral Science, University of Colorado Boulder, Boulder, CO;; d Department of Epidemiology and Biostatistics, Indiana University School of Public Health-Bloomington, Bloomington, IN;; e Department of Sociology, Woodrow Wilson School of Public and International Affairs, and Office of Population Research, Princeton University, Princeton, NJ;; f Harvard Center for Population and Development Studies, Harvard University, Cambridge, MA;; g Department of Sociology, the Ohio State University, Columbus, OH;; h Department of Sociology and Center on Health, Risk and Society, American University, Washington, D.C.;; i Department of Public Health, University of Missouri, Columbia, MO;; j Institute of Public Health, Faculty of Medicine, University of Heidelberg; and; k Department of Global Health and Population, Harvard T.H. Chan School of Public Health.

**Keywords:** HIV, incidence, older population, sexual behavior, South Africa

## Abstract

In South Africa, evidence shows high HIV prevalence in older populations, with sexual behavior consistent with high HIV acquisition and transmission risk. However, there is a dearth of evidence on older people's HIV incidence.

**Methods:**

We used a 2010–2011 cohort of HIV-negative adults in rural South Africa who were 40 years or older at retest in 2015–2016 to estimate HIV incidence over a 5-year period. We used Poisson regression to measure the association of HIV seroconversion with demographic and behavioral covariates. We used inverse probability sampling weights to adjust for nonresponse in 2015, based on a logistic regression with predictors of sex and age group at August 2010.

**Results:**

HIV prevalence increased from 21% at baseline to 23% in the follow-up survey. From a cohort of 1360 individuals, 33 seroconverted from HIV negative at baseline, giving an overall HIV incidence rate of 0.39 per 100 person-years [95% confidence interval (CI): 0.28 to 0.57]. The rate for women was 0.44 (95% CI: 0.30 to 0.67), double than that for men, 0.21 (95% CI: 0.10 to 0.51). Incidence rate ratios (IRRs) again show women's risk of seroconverting double than that of men (IRR = 2.04, *P* value = 0.098). In past age 60, the IRR of seroconversion was significantly lower than that for those in their 40s (60–69, IRR = 0.09, *P* value = 0.002; 70–79, IRR = 0.14, *P* value = 0.010).

**Conclusions:**

The risk of acquiring HIV is not zero for people older than 50 years, especially women. Our findings highlight the importance of acknowledging that older people are at high risk of HIV infection and that HIV prevention and treatment campaigns must take them into consideration.

## INTRODUCTION

HIV research, treatment, and prevention have mainly focused on populations younger than 50 years of age, with only a few studies highlighting the importance of the HIV epidemic in older age groups.^1–3^ For South Africa, home to the largest HIV epidemic worldwide, recent evidence shows high prevalence in older people^4,5^ and, in rural settings, sexual behavior in older people that is consistent with high acquisition and transmission risk.^6,7^ HIV prevalence is expected to increase at older ages because of South Africa's robust antiretroviral treatment (ART) program.^8^ However, HIV incidence at older ages is not well known. Earlier estimates of HIV incidence have used changes in prevalence^9^ or detection of recent infections through specific tests such as BED IgG-Capture Enzyme Immunoassay.^10^ Changes in prevalence are no longer good estimates of incidence because of longer survival and because the specific tests have not been applied in studies that include a sizeable sample of elders. Very few studies have used cohort data to establish HIV incidence in older populations.^5^ Longitudinal studies on HIV-negative, older populations that would allow for the direct measurement of HIV incidence are crucially needed.

This study uses a cohort of adults in rural South Africa who tested negative for HIV in 2010–2011 and were 40 years or older by the time of retest in 2015–2016 to estimate HIV incidence over an approximately 5-year period.

## METHODS

### HIV Cohort Creation: Surveys and Sample

Two waves of data on HIV infection were collected at the MRC/Wits Rural Public Health and Health Transitions Research Unit, where the Agincourt Health and Demographic Surveillance System (Agincourt HDSS) has been conducting regular update rounds of a 1992 baseline census. The Agincourt HDSS has been described in detail elsewhere.^11^ In sum, all households in the study site have been visited annually since 1992, with births, deaths, and migrations registered at each visit. Periodically, household, individual health, and sociodemographic data were also collected.

We calculated incidence from laboratory-based HIV tests of the same participants at 2 time points. The baseline study,^4^ the Ha Nakekela HIV and Noncommunicable Disease Study, ran from August 2010 to June 2011. It included an age-stratified and sex-stratified random sample of 7662 men and women aged 15 years and older drawn from the 2009 Agincourt HDSS census. Participants were asked to respond to a sexual behavior questionnaire and to an adapted WHO-STEPS questionnaire on noncommunicable diseases. We took anthropometric measurements, a point-of-care blood test for diabetes (CareSense) and lipid profile (CardioCheck), and dried blood spots (DBS) for HIV serostatus and viral load. HIV DBS testing was performed using screening assay Vironostika UniForm 11 (Biomerieux, France). All positive results were retested using the SD Bioline HIV ELISA test (SD; Standard Diagnostics Inc, Korea). Final reported results were determined as follows: If the screening test was negative, the result was reported as negative. If both the original result and the confirmatory retest were positive, the final result was positive. If the screening and confirmatory tests were discordant, a third assay (Elecsys, Roche, USA) was conducted. This third test determined the final result. If the final result was positive, the viral load was assessed using the Biomerieux NucliSens (Biomerieux, France) viral load assay.

We conducted a follow-up round. The Health and Aging in Africa: A Longitudinal Study of an INDEPTH Community in South Africa (HAALSI) study ran from November 2014 to November 2015, with a sample of 6281 women and men older than 40 years. The HAALSI sample was a random sample of individuals permanently living in the Agincourt study site during the Agincourt HDSS census of 2013 and all participants from the 2010 survey aged 40 years and older at the time of follow-up sampling.^12^ We conducted interviews to collect household and individual data that included socioeconomic data, self-reported health and specific diseases, anthropometric measurements, blood pressure, HIV status from DBS, and point-of-care blood tests that included glucose and lipid profiles. The HIV tests and diagnoses were performed in the same laboratory and followed the same methodology as the Ha Nakekela study.

Ethics clearance for the HDSS and for each survey was obtained from the University of the Witwatersrand Human Research Ethics Committee (Medical) and the Mpumalanga Provincial Research and Ethics Committee. The Ha Nakekela study also received ethical approval from the Institutional Review Board of the University of Colorado Boulder and the HAALSI study from the Harvard T.H. Chan School of Public Health, Office of Human Research Administration.

**FIGURE 1.**
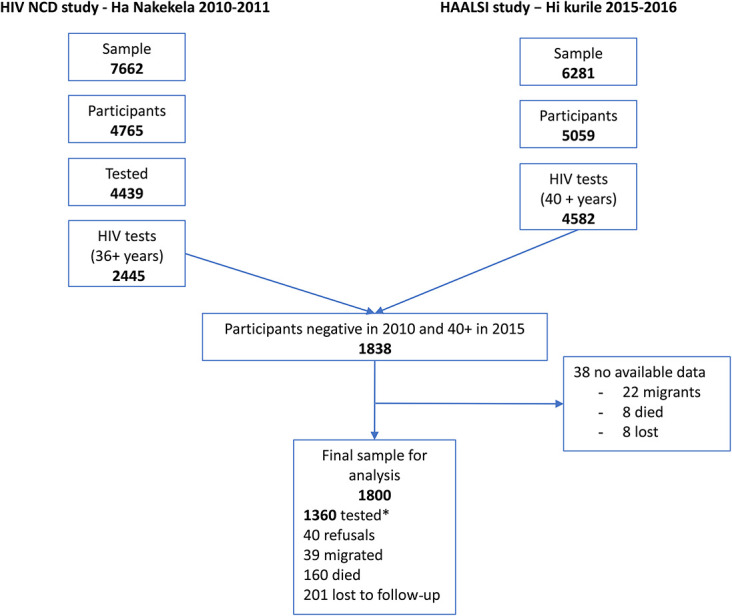
Creation of the HIV incidence cohort from the Ha Nakekela (2010) study and the HAALSI (2015) study in the Agincourt HDSS, rural South Africa.* Negative cases in 2010 that were part of the 2015 study.

Figure 1 shows the final cohort used to calculate HIV incidence rates (IRs). We focused on individuals who were 40 years or older at the second round and, therefore, 36 years or older in 2010–2011. In the first round, 2445 individuals aged 36 years and older consented to HIV testing: of which, 607 (25%) were HIV positive. Of those who were HIV negative, 38 had no further information in the HDSS. The remaining 1800 HIV-negative individuals became the sample for the second round; among these, 1360 (75.5%) individuals consented and were tested. This sample of 1360 comprises the cohort where we can directly measure HIV incidence because they were tested in both studies. Of the other 440 HIV-negative individuals at the first round, 40 (2.2%) refused to participate in the second round, 39 (2.2%) had migrated, 160 (9%) had died, and 201 (11.2%) could not be located for the second round.

### Statistical Analysis

We calculated HIV prevalence for the population aged 40 years and older for both surveys. For 5 years of follow-up, we calculated HIV incidence by age, gender, and other key sociodemographic characteristics. We used Poisson regression to measure the association of HIV seroconversion with demographic and behavioral covariates. We used inverse probability sampling weights to adjust for nonresponse in 2015, based on a logistic regression with predictors of sex and age group at August 2010. We estimated HIV incidence in 2 ways based on 2 estimates of the exposure time. The restricted estimate is based only on the 1360 people who were tested in the second round. For HIV-positive individuals at the second round, we assigned both time of infection and length of exposure as midway between the dates of the first and second tests. For HIV-negative individuals, exposure was the time between tests. We also calculated a conservative estimate using the full sample of 1800 people. For the 440 people who were not tested at the second round, we assigned exposure to be the time between the first test and death or outmigration if either of those events were observed or between the first test and the beginning of the HAALSI study for those who refused or were not found during the second round. This conservative estimate adds exposure time but no additional new infections, thereby providing a lower bound for HIV incidence. Those who aged 40 years during the follow-up time contributed exposure time only for the period when they were 40 years or older.

## RESULTS

HIV prevalence in those 40 years and older in the Ha Nakekela study in 2010 was 21% and increased to 23% 5 years later in the HAALSI follow-up survey.

Overall, 33 individuals tested at round 2 had seroconverted from HIV negative at baseline.

Table 1 shows IRs and IR ratios (IRRs) based on the conservative estimate of exposure and on Poisson regression of the association between incident HIV infection and key demographic variables. There were a total of 6753 person-years, giving an overall weighted HIV IR of 0.39 per 100 person-years [95% confidence interval (CI): 0.28 to 0.57], with a rate of 0.44 for women (95% CI: 0.30 to 0.67), double than that for men, 0.21 (95% CI: 0.10 to 0.51). HIV incidence was over 50% higher under the more restricted estimate of exposure (data not shown), with total person-years of 5537 and an overall HIV incidence of 0.60 per 100 person-years (95% CI: 0.43 to 0.84). Incidence in women 0.73 (95% CI: 0.50 to 1.07) was still double than that of men with 0.36 per 100 person-years (95% CI: 0.17 to 0.75).

**TABLE 1.**
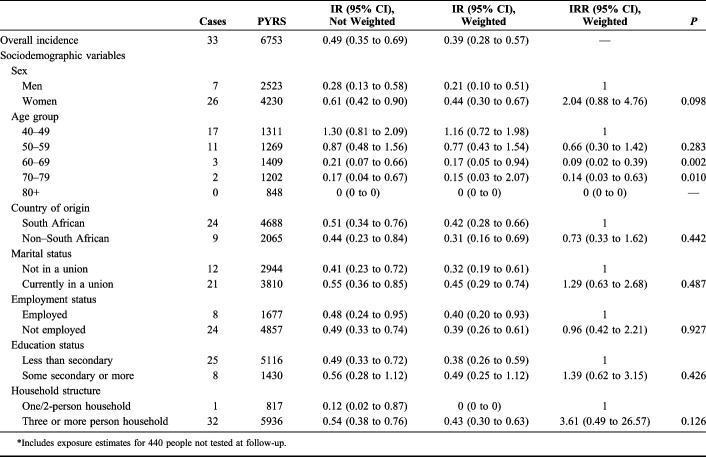
HIV IRs (Not Weighted and Weighted) and IRRs Per 100 Person-Years Over 5 Years of Follow-up (2010–2015) and Across Key Sociodemographic Covariates for a Cohort of 1800 Adults of Age 40 Years or Older at Follow-up; Conservative Estimate of Exposure*

The IRRs again show women's risk of seroconverting double than that of men (Table 1; IRR = 2.04, 95% CI: 0.88 to 4.76, *P* value = 0.098). Looking at age groups, only past age 60 had the IRRs of seroconversion significantly lower than that for those in their 40s and 50s (60–69, IRR = 0.09, 95% CI: 0.02 to 0.39, *P* value = 0.002; 70–79, IRR = 0.14, 95% CI: 0.03 to 0.63, *P* value = 0.010). There were no incident cases in people 80 years and older. We did not have enough cases to calculate sex-specific incidence by the age group. None of the sociodemographic variables analyzed (country of origin, marital status, employment status, education status, and household structure) were significantly associated with the risk of seroconversion (Table 1).

## DISCUSSION

To the best of our knowledge, this is only the second report of population-based HIV incidence in a cohort of 40 years of age and older in rural Africa, after Wallrauch et al^5^ who reported incidence in a population of 50 years of age and older in 2008. These results add to the existing evidence from rural South Africa that older, HIV-negative adults are at risk for acquiring HIV.^6,7,13–15^ Other studies in Africa have reported HIV incidence in younger age groups,^16–19^ using repeated measures in a voluntary counseling and testing (VCT) context,^20–22^ using mathematical models to estimate incidence from prevalence studies,^9,23,24^ or using the BED Capture Enzyme Immunoassay.^10^ These studies show an HIV incidence per 100 person-years ranging from 1.1 to 11.2, giving even more significance to the HIV incidence of 0.39 per 100 person-years found in the older population of our study. Our results show HIV seroconversion is still high even for those in their 50s and nonzero for those in their 60s and 70s. Disease acquisition seems to stop only over the age of 80 years. These results support previous studies on HIV prevalence in the Agincourt area, showing that even people in their 60s and 70s are at high risk of HIV mortality.^12,25^ Older age people in this area, although still reporting some sexual risk behaviors,^6,15^ are more likely to be in a regular union and to know the HIV status of their partners.^7^ Our incidence data, showing that women have higher levels of HIV infection than their male counterparts, seem to contradict the study by Wallrauch et al^5^ in which older women had lower risk of HIV than men. However, we believe that our results, produced 7 years after Wallrauch's and in a more advanced phase of the HIV epidemic in South Africa, may show the HIV epidemic trend where women, even at older ages, present higher vulnerability to HIV infection because of greater biological susceptibility, and lower sexual independence,^26^ and have higher risk of HIV seroconversion than men, as has been found at younger ages in South Africa^19^ and in Mozambique.^16^ In addition, our data do not support other findings that those with greater formal education have lower risk of HIV seroconversion.^27^

Because of the small number of individuals who seroconverted (n = 33) in the 5 years of follow-up, our power to show significant associations between incident HIV and sociodemographic factors was limited, and many of these estimates were measured imprecisely with wide CIs. However, we have previously described that this older population is sexually active and has high levels of sexual risk behaviors, such as low condom use and multiple partners.^6,7^

ART was gradually introduced into the study site from 2004, becoming fully available in 2010,^28^ around the time we did the baseline study. By the time of the follow-up study in 2015, 71% of HIV positives were on ART.^29^ In 2018, a national South Africa study found that 68% of HIV-positive people knew their status and were on ART.^30^

It is also important to consider death as a competing event for incident HIV infection. We identified 160 HIV-negative participants from the 2010 study (9% of all HIV negative), who died before the follow-up study. We do not expect AIDS-related deaths among those who tested negative in 2010 because the period between the 2 studies was only 5 years, whereas the life expectancy of those newly infected has been estimated to be between 8 and 10 years in the absence of treatment. This is also supported by Kabudula et al who saw a reduction of HIV mortality in the study area from 2008–2010 to 2011–2013.^25^ However, we might expect that those who died would have been less likely to seroconvert during follow-up (because of age or infirmity), had they not died. If this scenario held, even the conservative HIV incidence we observed would somewhat overestimate the true HIV incidence.

However, our results show the possible directions of the associations in this older, rural South African population and open the discussion on whether new HIV seroconversions would happen differently depending on age groups or African regions where there may be different sociodemographic risk factors.

This study is among the first to measure HIV incidence directly at the population level for an older population cohort. Despite the small size of the sample and the small number of conversions, our results clearly show that the risk of acquiring HIV is not zero for people older than 50 years. It is especially important that the risk is not zero for older women. Our findings highlight the importance of acknowledging that older people are at high risk of HIV infection and that HIV prevention and treatment campaigns must take them into consideration.
